# Substance used in the ripening of fruits can cause kidney damage and water and electrolytic disorders

**DOI:** 10.1590/2175-8239-JBN-2023-E009en

**Published:** 2023-11-03

**Authors:** Talita Rojas Sanches, Lúcia Andrade

**Affiliations:** 1 Universidade de São Paulo, Faculdade de Medicina, Departamento de Clínica Médica, Laboratório de Investigação Médica 12, São Paulo, SP, Brazil.

Calcium Carbide (CaC_2_) is a chemical substance used in the production of acetylene, steel and in welding^1^. Nonetheless, one way of using this substance is as an accelerator for ripening fruits for human consumption, especially with bananas^1^. The maturation of bananas and other fruits depends on intrinsic and highly coordinated processes in the fruit, especially if we consider that the maturation stage is the beginning of the senescence process^2,3^. Ethylene is the phytohormone responsible for initiating the maturation phase of climacteric fruits, such as bananas^2,3^. Due to demands from the food market, many producers choose to carry out the maturation of fruits artificially, in order to obtain volume and standardization of production^3^. CaC_2_ is a highly alkaline product, and in contact with water it produces calcium hydroxide (Ca(OH)_2_) and acetylene gas (C_2_H_2_). Acetylene gas is a substance analogous to ethylene, which mimics the function of the phytohormone, promoting standardized and rapid fruit maturation^1^. However, the use of CaC_2_ can produce a fruit with a ripe appearance, but with an immature interior, which leaves the fruit without the expected flavor^1^. More importantly, CaC_2_ can be a toxic substance, both for consumers and workers involved in fruit production^1–3^. CaC_2_ irritates the oral and nasal mucosae, in addition to causing gastrointestinal discomfort^1–3^. Acetylene gas is soluble in water and can buildup in fruits and affect the neurological system^1^. The impurities found in CaC_2_, such as traces of arsenic and phosphorus, can lead to acute irritation of the mouth and nose, vomiting, skin ulcers and even kidney damage^1,4^.

In an article published in the Brazilian Journal of Nephrology^5^, researchers from the University of Benin, Nigeria, reported that female rats fed bananas (*Musa paradisíaca*) artificially ripened with CaC_2_ showed electrolyte and renal alterations. Rats fed naturally ripened bananas showed no such changes. The authors observed a significant increase in plasma concentrations of bicarbonate and a decrease in plasma concentrations of potassium and chlorine in animals fed bananas exposed to CaC_2_ when compared to animals fed naturally ripened bananas. In addition, the renal function of these animals seemed to have been affected, with a significant increase in plasma urea and creatinine. These animals also showed alterations in the renal tissue, with glomerular atrophy and tubular necrosis. It is important to note that the serum levels of both chlorine and bicarbonate in the control groups were, interestingly, far below the normal range. Bicarbonate levels around 5 mEq/L and chlorine levels around 45 mEq/L. In any case, the animals fed with fruits ripened with CaC_2_ had bicarbonate levels significantly higher than those of the control groups, around 20 mEq/L, and chlorine levels significantly lower than those of the control groups, 30 mEq/L. Therefore, animals fed bananas ripened with CaC_2_ showed increased plasma bicarbonate (we cannot say that there was alkalosis, since bicarbonate levels were around 20 mEq/L), hypochloremia and hypokalemia. It is noteworthy that the number of animals per group was three.

As already mentioned at the beginning, CaC_2_ is extremely alkalizing in contact with water. Under normal circumstances, the kidneys excrete excess bicarbonate and restore acid-base balance^6^. Normal kidneys have an enormous capacity to excrete large amounts of bicarbonate when ingested chronically^7^. Failure of the kidneys to excrete excess bicarbonate is due to the presence of a mechanism that leads to the maintenance of metabolic alkalosis. The drop in glomerular filtration may explain the decrease in bicarbonate filtration. These animals may be presenting the calcium-alkali syndrome^8^. The calcium-alkali syndrome consists of the triad of hypercalcemia, metabolic alkalosis and acute kidney injury associated with the ingestion of large amounts of absorbable calcium and alkali^8^. The syndrome was originally described in association with the use of milk and sodium bicarbonate for the treatment of peptic ulcers^8^. Unfortunately, serum calcium levels were not measured. Hypochloremia could not be explained by the calcium-alkali syndrome. The presence of hypochloremia in metabolic alkalosis is justified by volume depletion and loss of hydrochloric acid by the stomach. However, there are no reports of vomiting in these animals.

Prohibited in several countries, the use of Calcium Carbide is still allowed in Brazil, although some cities have laws prohibiting its use. It is important to highlight that there are safe ways to manage the maturation of bananas, such as the method used by producers in Borborema, in the state of Paraíba, and described by Brazilian researchers from the State University of Paraíba and the Federal University of Campina Grande^3^. The researchers reported that the ripening of bananas using leaves of *Bowdichia virgilioides* Kunth, a plant popularly known as Sucupira-preto, was efficient and safe for consumption^3^. Thoroughly washing the fruits can help in the partial removal of CaC_2_.
